# Simple and effective sialography with modification of contrast injection

**DOI:** 10.1016/j.ijscr.2024.109626

**Published:** 2024-04-06

**Authors:** Rachmi Fauziah Rahayu, S. Yudhi Lillah, Kristanto Yuli Yarso, Dedy Chandra Hariyono, Monica Bellynda

**Affiliations:** a DepartementNeuroradiology, Head Neck Division, Deparment of Radiology, Moewardi General Hospital, Sebelas Maret University, Jl. Kol Sutarto No.132, Surakarta, Indonesia; b Department of Surgery, Oncology Division, Sebelas Maret University, Faculty of Medicine Indonesia, Jl. Ir Sutami No. 36a, Surakarta, Indonesia; c Deparment of Radiology, Moewardi General Hospital, Sebelas Maret University, Jl. Ir Sutami No. 36 a, Surakarta, Indonesia; d Department of Surgery, Moewardi General Hospital, Sebelas Maret University, Jl. Kol. Sutarto No. 132, Surakarta, Indonesia

**Keywords:** Sialocele, Sialography, Contrast, Injector

## Abstract

**Background:**

In several cases, a person can have an abnormal mass in the outer mandible or under the tongue and is usually accompanied with the decrease of saliva. Early and accurate examination is needed to diagnose this case. In this case report, we present two cases of salivary glands defect. The aim of this article is to submit two cases to review the etiology, risk factors, clinical manifestation, and examination methods of sialadenitis using a modified contrast injector to the duct of salivary gland.

**Case 1:**

A 17-year-old man came with a complaint of a lump under the right jaw. Sialography examination using a modified syringe with abbocath 24G showed an occlusion of the right and left Warton duct stoma. Right and left Bartholin's duct stoma occlusion post excision and marsupialization of the ranula. The complaint felt shrink after sialography. Three months follow-up, the patient said the lump was no longer felt and there were no complaints.

**Case 2:**

A 19-year-old man came with a complaint of clear fluid coming out when eating on the surgical scar under the right side of the jaw. From the plain photo, it appears that there is a missing amputatum of the right mandibular symphysis, body, angle, ramus processus condylaris et coronoideus of the left mandible. Sialography examination using a modified syringe with abbocath 24G showed suggest a sialocutaneous fistula (cut of the right mandibular ramus region to Stensen's duct and submandibular). Then the patient underwent fistula excision. A month follow-up after the excision patient had no feeling of lump but sometimes 3–4 drops still came out.

**Clinical discussion:**

Salivary tract injury is a quite rare case. Most frequent etiologies are iatrogenic. Sialography is a simple but effective method to identify obstruction of the salivary tract, including salivary tract injury. According to several studies, sialography identifies sialolithiasis with high sensitivity and specificity.

**Conclusion:**

Sialography is a simple but effective method that is beneficial for the treatment and examination with high specificity and sensitivity to assess the possibility of obstruction of the salivary ducts.

## Highlights

• Problems of the salivary glands have been outlined for centuries.
• Ultrasonography is the main choice of imaging for diagnosing salivary glands-related diseases.
• In the salivary glands duct examination, contrast injection using a modified tool is effective.
• Contrast injection duct could be solution for duct obstruction.

## 1 Introduction

Problems of the salivary glands have been outlined for centuries [1]. Obstruction is one of the problems that is more frequently caused by sialoliths, stricture/stenoses, mucus plugs [2], and retrograde infection of bacteria [1]. Stenosis of the salivary duct is a rare condition. However, 70–75 % of stenosis cases involve parotid glands. Salivary duct stenosis is the second most prevalent cause of obstruction in the parotid gland. Furthermore, stenosis can develop in over 13 % of patients after surgery of the gland or duct system area [3]. Stenosis can caused by salivary tract injury due to prior surgery [4]. Salivary tract injury can be traumatic or iatrogenic [5]. Based on Lewis and Knottenbelt's report, the incidence of parotid ductal injury is 0.21 % of trauma cases [6]. The surgery in the salivary gland area may have a salivary duct injury. The patient commonly experienced by the patient pain and decreased saliva, especially drooling. We can consider suspected salivary tract injury with these complaints that are felt postoperatively, most likely iatrogenic. Iatrogenicity due to previous surgical history is the most frequently reported cause in case reports of sialocele [7]. Sialocele formation may occur by surgical processes in the saliva or extravasation into the surgical defect that is delayed to form a fistula [8]. An early and accurate examination can increase the quality of life and determine the treatment that can be planned to overcome the complaint. Sialendoscopic treatment and dilatation show 85–95 % success without additional therapy. The promotion of serous saliva production is recommended through techniques such as gland massage, heightened hydration, and the use of sialagogues can improve recuperation [2]. Sialendoscopy is a harmless method for treating salivary gland obstruction [9]. This article is aimed to report the technique of examining suspected salivary tract injury using a modified injector to the duct of the salivary gland. This case has been written according to latest Surgical CAse REport (SCARE) Guide-lines [10].

## 2 Presentation of case 1

**Fig. 1:**
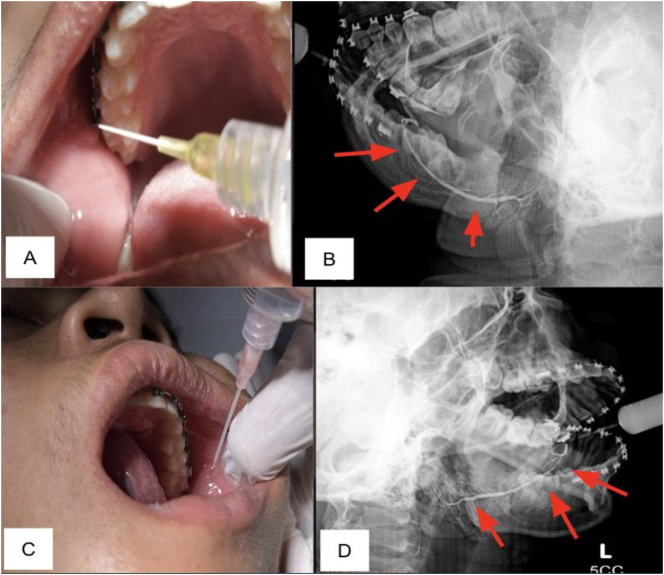
Contrast injection and imaging. A Contrast injection right side; B Right side of Stensen Duct, there is no obstruction; C Contrast injection left side; D Left side of Stensen Duct, there is no obstruction.

A 17-year-old man came with experience. The mass bellow right mandible comes and goes by itself for a month. There was no pain, swallowing disturbance, decreased appetite, or decreased weight. In September 2021, there was a 3 3-cm bluish mass filled with fluid below the tongue with no pain and lasted for three months. The patient was referred to the district hospital for further examination. At the hospital, the patient was diagnosed with ranulafour; then a ranula incision was made; within four weeks of follow-up, the complaints were reduced, and the surgical wound had healed. Four months later the patient returned to the hospital with complaints of feeling a lump a month after the last follow-up. The patient underwent a sialography. Sialography was then done using a modified injector with 24G abbocath, showing occlusion of the right and left stomal Wharton duct. The right and left stomal Bartholin duct occlusion due to excision and ranular manipulation was also found. There was no abnormality in the opening of the right and left Stensen duct (Fig. 1). The patient said the complaint felt shrink after sialography. Three months follow-up, the patient said the lump was no longer felt and there were no complaints (Fig. 4).

## 3 Presentation of case 2

**Fig. 2:**
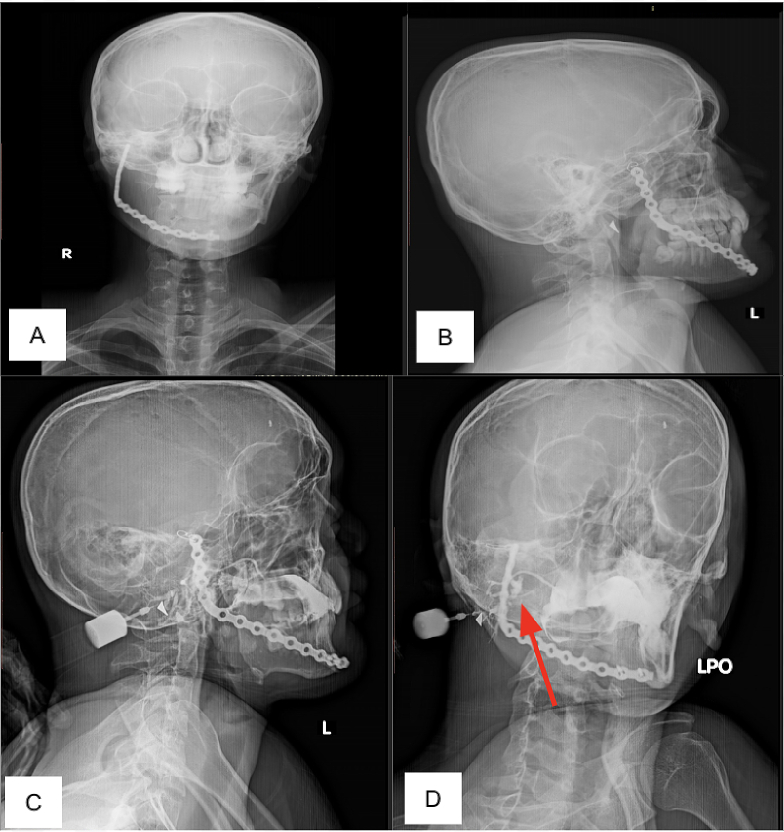
Plain and contrast rontgent. A Anterior plain rontgen; B Lateral side plain rontgen; C Anterior rontgen; D Lateral side plain rontgen red arrow pointing on fistula sialacutaneus. (For interpretation of the references to colour in this figure legend, the reader is referred to the web version of this article.)

**Fig. 3:**
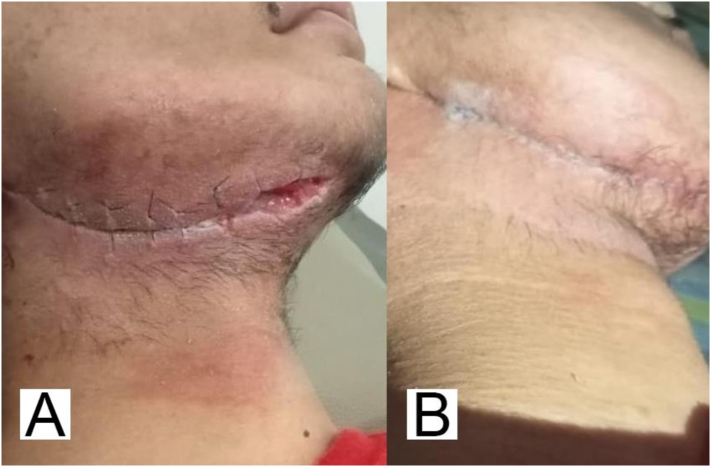
Postoperative wound dehiscence. A: Before repair and reconstruction procedures with wound dehiscence; B: Post repair and reconstruction with repaired wound dehiscence.

A 19-year-old man came with a serious fluid from the postoperative site under the right mandible whenever eating. A month before, the patient felt a mass in the right cheek that started appearing six months before. The mass is approximately as big as a chicken egg, with no pain, eating, or breathing disturbances. The patient is still able to open his mouth. The patient was first diagnosed with ameloblastoma in a type-C hospital, then referred to a type-A hospital to undergo surgery. At the referral hospital, an examination was done, and the diagnosis was ameloblastoma. The patient then planned to have a tumor resection and reconstruction by a surgical oncologist and plastic-reconstruction surgeon. The operation was carried out by resecting the tumor and then performing a right hemimandibulectomy and installing a 5 × 16 recon plate reconstruction. First-time patient post-operation follow-up, the patient has complained an imperfect wound closure, and the patient feels watery discharge when eating on the operation site. Physical examination found postoperative wound dehiscence below the right mandible with serous discharge, and no blood or pus. No pain was felt in scar palpation. A plain X-ray shows missing amputated from the right mandible symphysis, corpus, angulus, to ramus processus condylar et coronoideus of left mandible bone (Fig. 2). 14 cc contrast was then injected in 24G sabbath. Radiology finding shows a sial cutaneous fistula (cutan regio right ramus mandible to Stensen duct and submandibular). Then, the patient underwent fistula excision. A month follow-up after the excision, the patient had no feeling of a lump, but sometimes 3–4 drops still came out, and the patient did not feel pain or itching (Fig. 3).

**Fig. 4:**
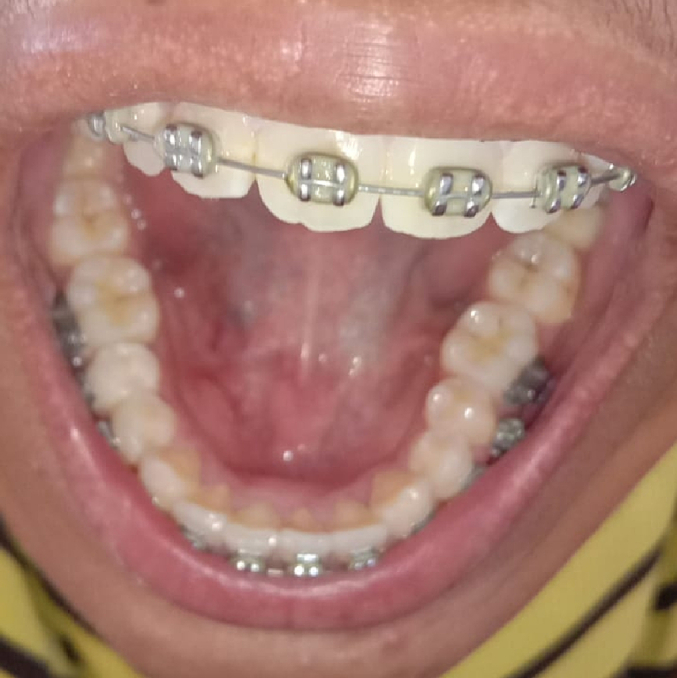
6-month follow up, there is no longer lump, the patient has no complaint.

**Fig. 5:**
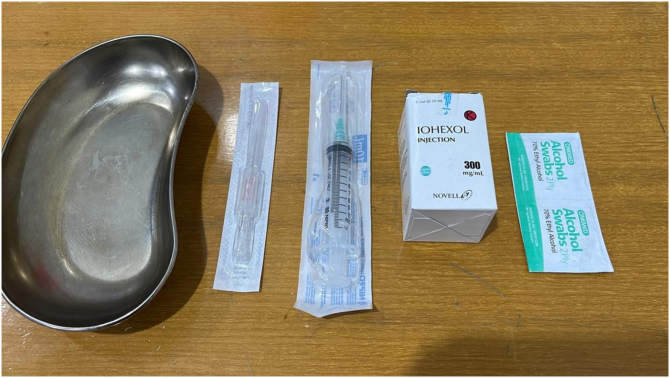
Sialography tools and materials.

## 4 Discussion

Epidemiologically, Salivary gland trauma is a rare occurrence [11]. Iatrogenic injury is the most common cause of parotid gland sialocele [12]. Salivary glands consist of submandibular gland, sublingual gland, and parotid gland [13]. In both cases, the sialocele occurred due to injury to the salivary tract after the previous surgical procedures. In the first case, there was occlusion, and in the second case, a sial cutaneous fistula was formed. The fistula formation in the second case was likely due to a surgical defect during the hemimandibulectomy with the recon plate installed. So that saliva, which is continuously secreted by the glands, enters and fills the space so that a cyst or fistula is formed, as in the article by Melville [8]. Materials in each of those glands will drain to the submandibular duct. Both right and left parotid glands will drain to the Stensen duct. The submandibular and sublingual gland flow the submandibular and sublingual glands will drain through the Wharton and Bartholin ducts [14].

Ultrasonography is the leading imaging method for diagnosing salivary gland-related diseases. Ultrasonography has limited accuracy in detecting stenosis in salivary ducts. The second choice of imaging is sialography, a classic method of examining ducts using contrast media that is still effective in picturing chronic inflammation of salivary glands ducts and sialolithiasis. Some research shows that sialography has high sensitivity and specificity for detecting sialolithiasis. The third imaging option is sialendoscopy, which is highly accurate for diagnosing salivary gland-related diseases, especially sialolithiasis. The procedure used in this case report was sialography, which has 86,7 % sensitivity and 71,4 % specificity and is considered as a simple choice with enough effectiveness in examining sialadenitis [15].

In this case report, classic sialography was done by referring to classic procedure of patient preparation before injecting contrast, which was using a stimulant or lime under the tongue 2 to 3 min before sialography, then followed by injecting contrast in the central duct (Fig. 3). The photo was then retaken to see the imaging of salivary glands. Ten minutes later, the contrast was cleaned [16]. A simple modification of split and 24G about was used in this case report (Fig. 5).

## 5 Conclusion

Contrast injection using a modified tool is effective in the salivary glands duct examination. The classical procedure of sialography has enough specificity and sensitivity to diagnose sialadenitis. A modified spuit and 24G abbocath tool split and 24G sabbath can be considered and it is effective in injecting contrast. Sialography is one of the salivary gland examination methods that is simple but effective with high specificity and sensitivity to detect salivary duct obstruction, including in cases of salivary gland injury.

## Consent

Patient Consent: Written informed consent was obtained from the patient for publication and any accompanying images. A copy of the written consent is available for review by the Editor-in-Chief of this journal on request. Parental Consent: Written informed consent was obtained from the patient's parents/legal guardian for publication and any accompanying images. A copy of the written consent is available for review by the Editor-in-Chief of this journal on request.

## Provenance and peer review

Not commissioned, externally peer-reviewed.

## Ethical approval

Ethical approval for this study (814/VI/HREC/2023) was provided by the Health Research Ethics Comitte on 15 September 2023.

## Funding

This research did not receive any specific grant from funding agencies in the public, commercial, or not-for-profit sectors.

## Author contribution

Yudhi Lillah Setyawati, Rachmi Fauziah Rahayu, Kristanto Yuli Yarso: study concept, imaging for this patient, surgical therapy for this patient.

Yudhi Lillah Setyawati: Data Collection, Writing Original draft preparation.

Rachmi Fauziah Rahayu, Kristanto Yuli Yarso: Senior author and the manuscript reviewer.

Kristanto Yuli Yarso: Guarantor and Coresponding author.

Yudhi Lillah Setyawati, Monica Bellynda: Editing – Writing.

All authors read and approved the final manuscript.

## Guarantor

Kristanto Yuli Yarso, Yudhi Lillah Setyawati.

## Research registration number

Not applicable. This is not a clinical trial or ‘First in Man’ study.

## Conflict of interest statement

The authors declare no conflict of interest.
